# Automated identification of brain tumors from single MR images based on segmentation with refined patient-specific priors

**DOI:** 10.3389/fnins.2013.00241

**Published:** 2013-12-17

**Authors:** Ana Sanjuán, Cathy J. Price, Laura Mancini, Goulven Josse, Alice Grogan, Adam K. Yamamoto, Sharon Geva, Alex P. Leff, Tarek A. Yousry, Mohamed L. Seghier

**Affiliations:** ^1^Wellcome Trust Centre for Neuroimaging, University College of LondonLondon, UK; ^2^Departamento de Psicología Básica, Clínica y Psicobiología, Universitat Jaume ICastellón, Spain; ^3^Lysholm Department of Neuroradiology, National Hospital for Neurology and NeurosurgeryLondon, UK; ^4^Hôpital de la Pitié-Salpêtrière, Institut du Cerveau et de la Moëlle épinièreParis, France; ^5^Developmental Cognitive Neuroscience Unit, Institute of Child Health, University College of LondonLondon, UK; ^6^Institute of Cognitive Neuroscience, University College of LondonLondon, UK

**Keywords:** automatic lesion identification, segmentation, spatial normalization, fuzzy clustering, MRI

## Abstract

Brain tumors can have different shapes or locations, making their identification very challenging. In functional MRI, it is not unusual that patients have only one anatomical image due to time and financial constraints. Here, we provide a modified automatic lesion identification (ALI) procedure which enables brain tumor identification from single MR images. Our method rests on (A) a modified segmentation-normalization procedure with an explicit “extra prior” for the tumor and (B) an outlier detection procedure for abnormal voxel (i.e., tumor) classification. To minimize tissue misclassification, the segmentation-normalization procedure requires prior information of the tumor location and extent. We therefore propose that ALI is run iteratively so that the output of Step B is used as a patient-specific prior in Step A. We test this procedure on real T1-weighted images from 18 patients, and the results were validated in comparison to two independent observers' manual tracings. The automated procedure identified the tumors successfully with an excellent agreement with the manual segmentation (area under the ROC curve = 0.97 ± 0.03). The proposed procedure increases the flexibility and robustness of the ALI tool and will be particularly useful for lesion-behavior mapping studies, or when lesion identification and/or spatial normalization are problematic.

## Introduction

Meaningful lesion-behavior mapping in patients with focal brain damage (Bates et al., 2003; Rorden et al., 2009) rests upon precise identification of each patient's lesion. The most commonly used method for lesion identification is manual tracing of abnormal brain tissue by trained experts; this is conventionally taken to be the gold-standard method. However, the manual tracing of lesions is laborious, time-consuming and largely operator-dependent (Fiez et al., 2000). To overcome these limitations, several automated procedures for lesion identification have been proposed (see review in Gondal and Khan, 2013; Gordillo et al., 2013), but they generally involve multi-spectral brain images (e.g., Soltanian-Zadeh et al., 1998; Capelle et al., 2004; Prastawa et al., 2004; Wu et al., 2006b; Kabir et al., 2007; Ruan et al., 2007; Corso et al., 2008; Nie et al., 2009; Menze et al., 2010; Hsieh et al., 2011; Popuri et al., 2012). The acquisition of 3D multi-spectral anatomical images is not always possible due to financial or time constraints. Indeed, it is not unusual that only one anatomical image (usually a T1-weighted image) is acquired from patients participating in research fMRI studies, particularly when total scanning time over fMRI runs exceeds 60 min. In these cases, tumor identification needs to be based on the anatomical images available. The aim of the present study is to demonstrate the usefulness of an automated method for identifying brain tumors from single T1-weighted images.

**Figure 1:**
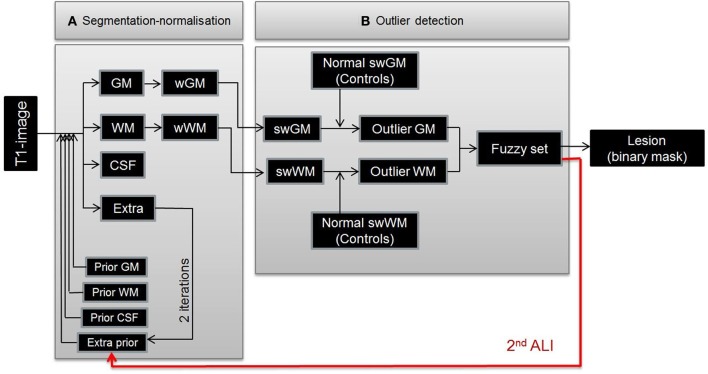
**Illustration of ALI procedure. The method identifies lesion in two core procedures. (A)** A modified segmentation-normalization that identified the likelihood that each voxel belonged to one of four different tissue types and **(B)** a fuzzy clustering algorithm that identified voxels where the tissue probability maps differed from those in healthy controls. The red arrow shows the recursive nature of the procedure for the tumor identification. The first iteration of steps **(A)** and **(B)** (1st ALI) generated a first approximation of the tumor site. The second iteration of steps **(A)** and **(B)** (2nd ALI) included the approximate lesion definition (see red arrow) as a patient-specific prior to improve the tissue segmentation. (w, normalized; sw, smoothed and normalized).

We previously proposed an automated lesion identification (ALI) method that can operate on a single anatomical image (Seghier et al., 2008, see Figure 1). This procedure defines a lesion as a set of atypical voxels identified as outliers in gray and white matter tissue images that resulted from a whole-brain segmentation into a standard reference space (MNI). This method rests on two innovations: (A) optimization of the unified segmentation-normalization algorithm (Ashburner and Friston, 2005) with the addition of an extra “tissue class prior” that explicitly models the presence of atypical tissue, and (B) a fuzzy clustering outlier detection procedure that identifies atypical tissue in both normalized gray and white matter, by comparing the patient's tissue image, voxel-by-voxel, against a group of normal subjects' tissue images. The ALI method has already been shown to have high sensitivity to brain lesions in MRI images acquired from patients with stroke (Seghier et al., 2008). Our aim here is to develop the procedure for the detection of brain tumors. Other approaches have been proposed to delineate brain damage from only one type of MRI contrast image, but they were not developed specifically for brain tumors (e.g., multiple sclerosis infarct lesions or cortical malformations, see Shen et al., 2008, 2010; Thesen et al., 2011; Lladó et al., 2012), operate on 2D slices only (Xu and Mandal, 2012), or require contrast enhanced images that are not commonly acquired in research studies (Assefa et al., 2010; Harati et al., 2011; Asman et al., 2013).

**Figure 2:**
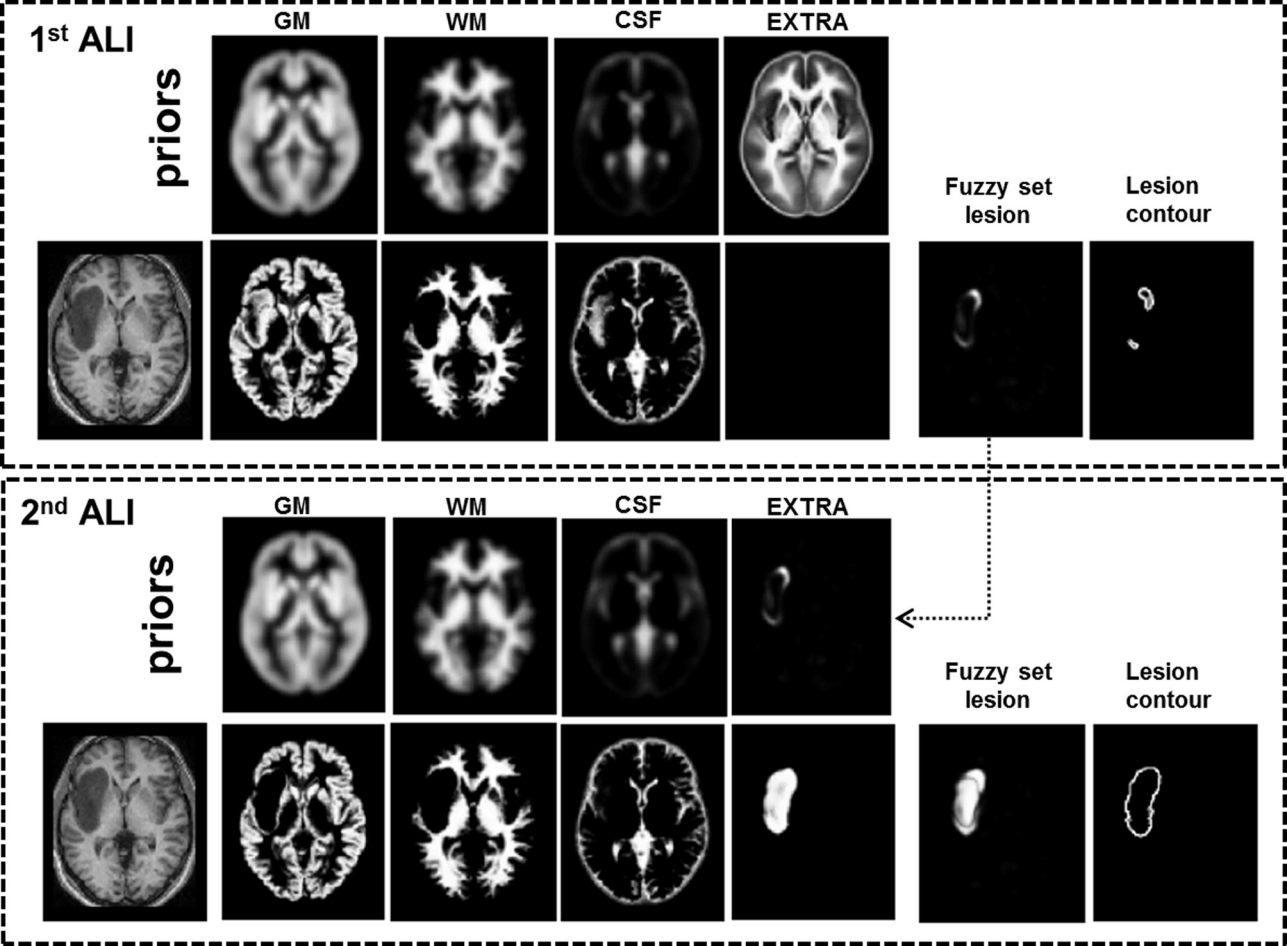
**Illustration of our recursive ALI procedure for patients with brain tumors.** With standard ALI (**Top** panel), the lesion is identified in the correct location, but not the total extent (see fuzzy set, lesion contour, and empty extra prior), because the segmentation step has misclassified some voxels in the lesion as normal GM or WM. This problem is resolved when the fuzzy set from the 1st ALI run is used as the extra prior in the 2nd ALI run (**Bottom** panel).

Brain tumor identification can be challenging for tissue segmentation procedures. Apart from their diversity in shape, size, and location, the presence of a tumor in brain images can be associated with a highly heterogeneous and diffuse signal distribution that can be confounded by a similar range of signal intensity in the neighboring intact tissue (Veloz et al., 2011). Indeed, our initial analysis using the original implementation of ALI (Seghier et al., 2008) was able to identify the correct location of the tumors but was not always able to delineate their full extent. This was driven by misclassification of atypical tissue (i.e., tumor) as gray or white matter during the segmentation step, despite the inclusion of an extra tissue class prior in the segmentation procedure (see Figure 2, top panel). To enhance its accuracy, we propose here a practical solution that uses ALI in a recursive mode: a first run of ALI provides an approximation of the tumor size and location, and a second run of ALI includes that approximation as a patient-specific “prior” to model the extra tissue class (See Figure 2, bottom panel). This practical solution that incorporates a refined patient-specific tissue class prior in the final segmentation of the patient's brain image, significantly improved the accuracy of tumor identification. Below, we illustrate how the automated procedure deals with real lesions, using datasets from a heterogeneous sample of 18 patients with brain tumors. The automatically identified tumors were compared to manual segmentations delineated by two independent observers.

## Materials and methods

The study was approved by the London Queen Square Research Ethics Committee. All subjects gave written informed consent prior to scanning.

### Data sets

*T1*-weighted images of 18 patients (8 females, mean age: 37.3 years, age range: 16–66 years) with brain tumors, acquired as part of an fMRI language presurgical evaluation, and 64 neurologically normal subjects (age range: 21–75 years) were included in this study. The heterogonous sample of patients consisted of 9 patients with low grade gliomas (World Health Organization (WHO) grade I: 1 dysembryoblastic neuroepithelial tumor; WHO grade II: 2 oligoastrocytomas, 3 oligodendrogliomas, 1 astrocytoma and 2 diffuse astrocytomas), 8 patients with high grade gliomas (WHO grade III: 3 anaplastic astrocytomas, 1 glioneuronal tumor with neuropil-like islands, 1 diffuse astrocytoma with anaplastic progression; WHO grade IV: 3 glioblastomas) and one meningioma. Tumors varied in size and location, in both hemispheres, as listed in Table 1. Overall, tumors were predominately located in language and motor regions, with lesion size varying between 1.5 and 23 cm^3^ (Table 1). It is worth noting that multispectral data are typically used for tumor segmentation, in particular in the clinical setting (Gordillo et al., 2013), namely to provide complementary information about the different tumor parts; however, we do not have multispectral data in all our 18 patients.

**Table 1:**
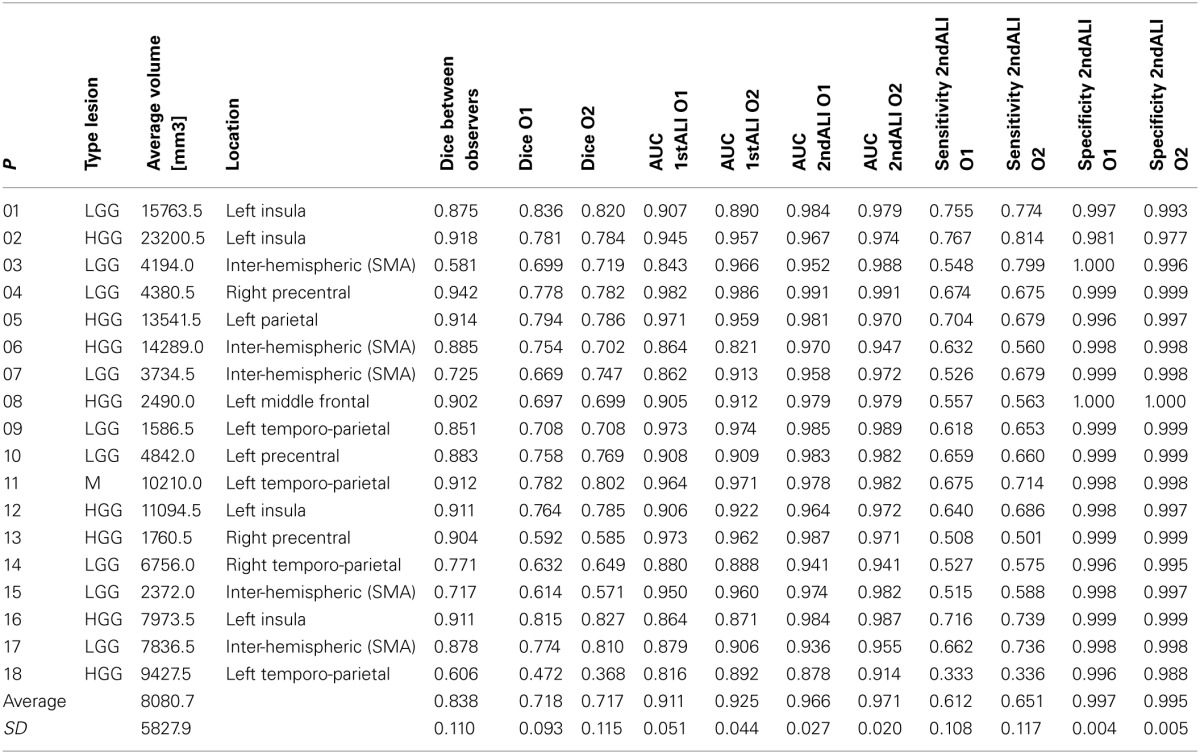
**Relevant lesion information (type, size, and tumor location) and similarity measures (Dice and AUC)**.

Dice's similarity index is shown for the comparison between (1) the manually-segmented tumors of the observers and (2) each binary lesion map obtained with the recursive ALI (i.e., fuzzy sets at a U threshold of 0.2) and the manually-segmented tumors of each observer. The area under the curve (AUC) was obtained for the comparison of the manually-segmented tumors of each observer and the binary mask obtained for different U thresholds from both the standard (1st) and recursive (2nd) ALI approaches. Finally, the specificity and sensitivity of the recursive ALI (at a U threshold of 0.2) are also reported. (P, Patient; LGG, Low Grade Glioma, HGG, High Grade Glioma; M, Meningioma; O1, Observer1; O2, Observer2 and SMA, Supplementary Motor Area).

### MRI data acquisition

Structural data consisted of high-resolution T1-weighted MRI scans acquired on a Siemens 1.5 T Sonata scanner (Siemens Medical Systems, Erlangen, Germany) using a 3D modified driven equilibrium Fourier transform sequence (Deichmann et al., 2004), consisting of 176 sagittal partitions with an image matrix of 256 × 224 and a final isotropic resolution of 1 mm^3^ (TR/TE/inversion time = 12.24/3.56/530 ms).

### Lesion identification procedure

The present paper uses ALI in the specific context of brain tumor identification. Below we explain how ALI works and how it can be used, in a recursive mode, to refine the definition of each patient's tumor in an informed way.

#### The automated lesion identification (ALI) method

ALI was initially implemented as a toolbox in the SPM software package (Wellcome Trust Centre for Neuroimaging, London, UK) to specifically deal with lesion identification on mono-spectral MRI scans. It comprises (A) segmentation-normalization and (B) outlier detection in the following steps (see Figure 1, cf. Seghier et al., 2008):

- A modified implementation of the unified segmentation-normalization procedure of SPM as described in Ashburner and Friston (2005). It combines segmentation, bias correction and spatial normalization through the inversion of a single unified model. Its algorithm is based on Bayesian statistics that allow previous knowledge (i.e., priors) to be incorporated. In the context of patients with brain lesions, the standard unified segmentation-normalization tends to misclassify the abnormal tissue as “normal” gray matter (GM), white matter (WM), or cerebral spinal fluid (CSF) because there is no explicit model of what constitutes abnormal tissue. The solution to this problem, as implemented in ALI, is to explicitly “inform” the segmentation procedure that abnormal tissue might be expected, (for a similar rationale see Rouainia et al., 2006; Menze et al., 2010). Practically, this entails adding an extra tissue class prior (known as the “extra class”) into the segmentation procedure to enable abnormal voxels to be modeled explicitly. Initially, the mean of WM and CSF priors provides an initial estimate of the extra tissue class prior, which is then iteratively optimized at the individual patient level (as detailed in Seghier et al., 2008) to provide a more accurate tissue segmentation with minimal misclassification. The output is a set of 4 normalized and segmented images per subject that code the probability that each voxel belongs to a particular tissue class (i.e., GM, WM, CSF and the extra class). In patients with damage to GM and WM, the damaged tissue is assigned to the extra class rather than the GM or WM images. Consequently, the probability that this tissue is GM or WM is reduced relative to normal at the site of the lesion. As we describe in the next step, the lesion can therefore be identified as reduced GM or WM relative to normal.
- Abnormal voxels in GM and WM are identified by an outlier detection algorithm according to the fuzzy logic clustering principle (cf. Seghier et al., 2007). The rationale is that, in the context of lesion identification, lesioned brain tissue is assumed to be an outlier vis-a-vis the normal range across the control group. To identify the atypical tissue (i.e., lesion), we apply the following steps: (1) the segmented and normalized GM and WM segments of each subject (patient and controls) are spatially smoothed to account for inter-subject anatomical variability (the default Gaussian kernel of 8 mm FWHM was used here); (2) GM and WM lesion images, as fuzzy sets, are created by comparing the smoothed GM and WM segments of the patient to those of the healthy controls. These lesion images code the degree of abnormality “U,” computed here as a degree of membership (as detailed below), varying between 0 to 1 at each voxel; and (3) the resulting GM and WM lesion images are combined to obtain a single “fuzzy set” (i.e., a lesion image) that fully defines the degree of abnormality at each voxel of the brain. Following fuzzy logic rules (e.g., Ibrahim, 1996), the latter is done using the operator “max” over all voxels of the GM and WM lesion images. This fuzzy set can be thresholded at a given *U*-value to generate a 3D-binary image of the lesion. Given the difficulty in defining an objective universal threshold that works on any dataset, we previously recommend that U thresholds are typically set at around 0.3 to provide accurate lesion delineation when ALI is used on images from chronic stroke patients (Seghier et al., 2008).

Below is a succinct description of the outlier detection algorithm used in ALI. The core of its algorithm is a non-iterative Fuzzy Clustering with fixed Prototypes (FCP) method previously introduced by Seghier and colleagues (2007). In contrast to standard data-driven fuzzy clustering techniques, FCP is a hypothesis-driven technique where the number and center of the clusters (i.e., prototypes) are predefined (i.e., number of clusters = number of subjects). By assuming that a lesioned brain is an outlier in relation to normal (control) brains, FCP can look for voxels that are very different in the lesioned brain as compared to controls.

The algorithm of FCP exploits the well-known fact that regression analysis is sensitive to outlier values. First, the centroids of the clusters/classes are predefined as following:

$$V_{kj}=\{\begin{array}{cc} \alpha & k=j\\ 0 & k\neq j \end{array}$$

Therefore, the centroid of the *j*-th cluster [with *j* = 1…*N*_sub_; *N*_sub_ is the total number of subjects (i.e., number of controls plus the patient)] is a vector of zeros except the *j*-th value which is set to α (with α ≠ 0). The real constant α is a “tuning” parameter that (1) adjusts the sensitivity of the method to outlier values, and (2) its sign determines whether to look for abnormally high (α > 0) or low (α < 0) outlier values; here α is fixed at −0.5, cf. Seghier et al. (2008).

We then quantify a similarity metric *D*_*ij*_ at each voxel *i* of the brain of the *j*-th subject:

$$D_{ij}=1-\tanh\left(\frac{N_{sub}}{\alpha^{2}}\cdot cov(P_{i},V_{j})\right)$$

*tanh* is the hyperbolic tangent, *cov* is the covariance between *P*_*i*_ (a vector of tissue probability at voxel *i* across all subjects) and *V*_*j*_ (the centroid of the *j*-th cluster). The distance *D*_*ij*_ lies in the range zero to two and will be small when *P*_*i*_ covaries highly with *V*_*j*_; this means the effects in voxel *i* is driven or influenced by the *j*-th subject. The above formula can be rewritten as follows (cf. Seghier et al., 2007):

$$D_{ij}=1-\tanh\left(N_{sub}\cdot \frac{\bar{P_{i}^{+j}}-\bar{P_{i}^{-j}}}{\alpha }\right)$$

Where $\bar{P_{i}^{-j}}$ is the mean of the tissue probability at voxel *i* over all subjects excluding the *j*-th subject, and likewise $\bar{P_{i}^{+j}}$ is the mean of the tissue probability at voxel *i* over all subjects including the *j*-th subject. Accordingly, at a given voxel *i*, *D*_*ij*_ is a measure of (1) how far subject *j* is from the group mean, and (2) how the group mean is perturbed when subject *j* is included. This observation is important and suggests that the FCP algorithm is formally similar to regression diagnostic methods that assess the extent to which a particular data point influences the model, by determining the change when that point is omitted (see a detailed discussion in Seghier et al., 2007).

The similarity metric *D*_*ij*_ is then used to quantify the degree of membership *U*_*ij*_ of voxel *i* to cluster *j* as follows:

$$U_{ij}=\frac{D_{ij}^{\lambda }}{\sum_{j}D_{ij}^{\lambda }}$$

Where the real constant λ is a “defuzzification” parameter: when λ tends to-∞ the classification becomes crisp and *U*_*ij*_ takes the value 0 or 1, but when λ goes to 0 the classification is fuzzy (*U*_*ij*_ is near to 1/*N*_*sub*_). We held λ constant at −4 (cf. Seghier et al., 2007, 2008).

When *j* indexes the patient, the set of *U*_*ij*_ values comprise a GM (or WM) lesion image, which represents the degree of membership (i.e., abnormality) of voxels that have very low GM (or WM) probability in the patient relative to controls. Both GM and WM lesion images are then combined to obtain a single lesion image (as a fuzzy set) that codes the degree of abnormality at each voxel of the brain.

#### Recursive ALI

When ALI was tested with its default parameterization on patients with brain tumors, it identified some tumor tissue at the correct location, but not always with the correct extent. Specifically, in those cases, the segmentation step with the default extra prior failed to detect all the atypical tissue, and hence misclassified tumor voxels as either intact GM or WM (see Figure 2, top panel). The outlier detection algorithm (step “B” as detailed above) cannot recover the misclassified tumor voxels and thus we needed first to ensure accurate brain segmentation (step “A”) in the presence of tumors. This can be achieved by an optimization of the priors used to constrain tissue classification. One obvious possibility was to increase the number of iterations (i.e., number of segmentation runs in step “A”). This is because the prior of the extra class is optimized iteratively in the segmentation step of ALI (see Figure 1); hence more iterations could theoretically increase the extent of the abnormal tissue in the extra tissue class. However, this was not the case with some of the images from patients with tumors because the extra prior was empty (or including few voxels with very weak probabilities) after the first iteration, making any additional iterations to the segmentation step ineffective.

Our solution to this problem, in the segmentation step, was motivated by the fact that, in a Bayesian sense, posterior estimates of the tissue classes improve with the accuracy of the definition of the spatial priors (see for example Levy-Cooperman et al., 2008). As mentioned above, we first noticed that tumors were being identified at the correct location with ALI, but not always with the full extent. By using ALI in a recursive mode, we assumed that the output from a first run of ALI would serve as a good empirical definition of the prior for the extra class (See Figure 1, red arrow). ALI could then be repeated with the same parameters but with the inclusion of a refined patient-specific prior for the extra class that corresponded to the fuzzy set lesion image obtained during the first run (see Figure 2, bottom panel). In other words, we suggest a “hierarchical” framework where the output from a first run of ALI serves as a prior in a second (or more) run of ALI. This extra prior is expected to grow nonlinearly with the number of iterations in the segmentation step in order to encompass the full extent of the lesion (as detailed above). Here we assume that two repetitions of ALI is near-optimal for our purposes. Conceptually, as reported in Cardoso et al. (2011), the aforementioned estimated priors are not proper priors from a mathematical standpoint, as they are derived from the data (i.e., generated by transforming the posteriors after the first run of ALI). However, they act as proper priors in the segmentation step of the second run of ALI. Below we show how running ALI in a recursive mode significantly improved the lesion definition in challenging cases while maintaining the automatic nature of the procedure.

### Validation

The outputs from our recursive ALI procedure were compared to the gold standard: manual segmentations of the same tumors. Practically, outline of the lesion was drawn on the outer borders of abnormally intense regions. The lesions were identified on a slice by slice basis on the T1-weighted images in the native space using MRIcron software (at http://www.mccauslandcenter.sc.edu/mricro/). Abnormal gyri were included in the lesion definition when there was a clear asymmetry in gyrus width between the lesioned and non-lesioned hemisphere, excluding areas of abnormality far from the lesion that could be related to mass effects. Periventricular regions were defined as lesioned only when there was a clear signal intensity change in the area and the cortical lesion extended all the way to the periventricular space. Areas surrounding enlarged ventricles with normal signal intensity, or periventricular white matter changes appearing on both hemispheres, were not defined as lesioned. This laborious task (requiring 1–5 h per brain depending on tumor size) generated binary lesion definitions in the individual native space of each patient. The binary definitions of the manually-segmented tumors were subsequently transformed into the MNI space using the normalization parameters generated from the segmentation step of ALI. Here, the manual segmentation was performed by two independent trained observers (authors AS and SG) in order to appreciate the inter-rater variability (for a similar rationale see Fiez et al., 2000). The first observer (AS) has good neuroanatomical knowledge but with less than 20 h of manual tracing experience. The second observer (SG) is an experienced operator who is familiar with lesion-tracing on MRI scans (see Geva et al., 2012).

We examined both the global (qualitative) and voxel (quantitative) levels to validate our procedure. At the global level, the method is successful if visual inspection confirms that the tumor is identified at the right location and with relatively accurate boundaries. This is the approach normally adopted when manual segmentation is used. At the voxel level, we quantified the similarity between the results of ALI and the results of the manual segmentation of the same tumors using different measures: The Dice's similarity Index and the receiver operating characteristic (ROC) curve.

The Dice's similarity index (Dice, 1945) was used to estimate the similarity between two binary images using the following formula:

$$\text{Dice}=\frac{2TP}{2TP+FP+FN}$$

Where the *TP*, *FP*, and *FN* represent the number of true positives (TP), false positives (FP) and false negatives (FN) in one approach relative to another. We calculated the Dice's similarity index for (1) each binary lesion map of the automated procedure (i.e., fuzzy sets at a given U threshold) relative to the manually-segmented tumors of each observer, and (2) the manually-segmented tumors of one observer relative to that of the other. The higher the Dice's index (ranging from 0 to 1) the higher the similarity between lesion definitions.

We also wanted to evaluate the sensitivity and specificity of our automated procedure in comparison to the manual segmentation of the same tumors. The sensitivity indicates the percentage of correctly classified voxels within the tumor (true positive rate), and the specificity represents the proportion of correctly classified non-tumor voxels (true negative rate). To estimate these values, we generated ROC curves (e.g., Metz, 1978) that encode the dependence of the true positive rate (sensitivity) on the false positive rate (one minus specificity) for different U thresholds. Perfect tumor segmentation would yield a point in the upper left corner of the ROC curve (i.e., no false negatives and no false positives). A completely random segmentation would result in a value along the diagonal line of the ROC curve. The area under the curve (AUC) (Hanley and McNeil, 1982) was assessed as a measure of the effectiveness of the recursive ALI method. Although the interpretation of the AUC might be problematic, particularly when comparing between models or classifiers (Lobo et al., 2008; Powers, 2012), it has nevertheless been widely used for assessing the accuracy of automated segmentation methods. Here, we provided both AUC values and Dice similarity indices in all patients as previously recommended (for a discussion see Zou et al., 2004). For completeness, the results were also reported in terms of sensitivity and specificity. Last but not least, AUC was obtained for both the standard and recursive ALI approaches, and a Wilcoxon signed rank test was used to assess whether the AUC values for our recursive approach were statistically larger than those obtained with the standard ALI.

## Results

**Figure 3:**
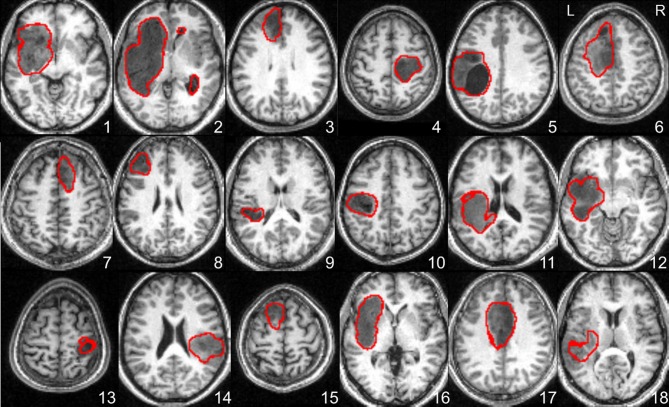
**Axial slices illustrating the lesion boundaries in each of the 18 patients as defined by the recursive ALI procedure detailed in Figures 1, 2.** The tumor contour was obtained with a threshold of *U* > 0.3. L, Left; R, Right.

At the global (visual inspection) level, our recursive ALI procedure was able to detect tumors at the right location and with the correct extent in all cases, except one (Patient 18). The boundaries identified for each tumor are shown in Figure 3 which demonstrates that the recursive ALI procedure successfully identified tumors of different types (low and high grade gliomas) and sizes, including tumors located in the insula (e.g., see Patients 1 and 16), close to the inter-hemispheric fissure (e.g., Patients 6 and 17) or near to the ventricles (e.g., Patient 11).

**Figure 4:**
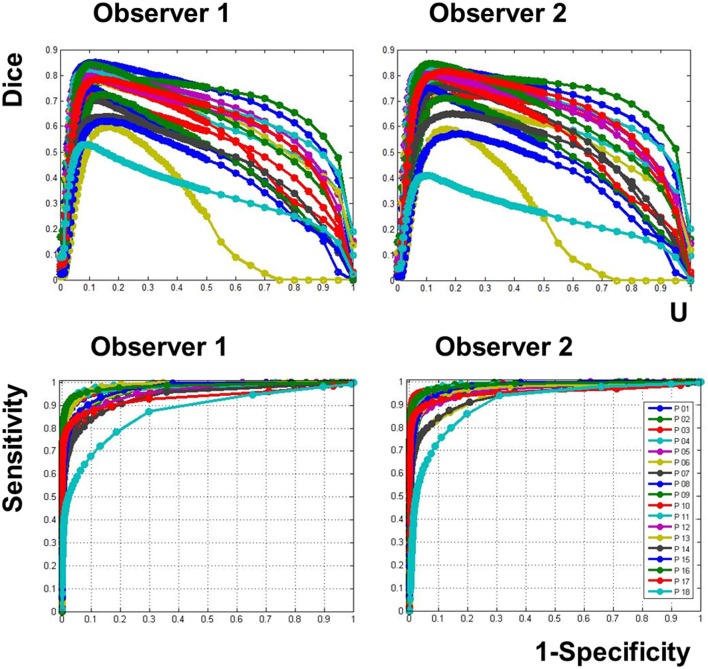
**(Top Panel)** Dice's similarity index when the binary lesion from the recursive ALI procedure is compared to each observer's manual segmentation (left = Observer 1; right = Observer 2), across a range of different U thresholds used to convert the fuzzy image into a binary image. The different colors represent different patients. **(Bottom panel)** ROC curves for different U thresholds. All the curves are close to the top-left corner (near to the manual segmentation). Patient 18 (with the most challenging tumor definition) is displayed in turquoise.

At the voxel level, the Dice's similarity index between the two observers was on average 0.838 (±0.110). Figure 4A illustrates the similarity, according to the Dice index, between each binary tumor image obtained with our recursive ALI method compared to the manually-segmented tumors. For each patient, the Dice similarity index was generated multiple times across a range of different U thresholds used in the conversion of the fuzzy image into the binary image. The plots in Figure 4A illustrate that similarity with the manual segmentation approach was highest (higher Dice indices) when the threshold on the degree of abnormality U ranged from 0.1 to 0.3. The ROC curves for all patients showed that our method is highly sensitive and specific in comparison to manual segmentation (see Figure 4B). The AUC from the ROC analysis for the recursive ALI was 0.97 on average (range = 0.90–0.99), which suggests an excellent agreement with the manual segmentation. It was also significantly higher (Wilcoxon signed rank test: *z*-value: 3.7, *p* < 0.001) when compared with the standard ALI (mean *AUC* = 0.92; range = 0.84–0.98). See also Table 1 for a list of all values for each individual patient including the Dice similarity index (at a U threshold of 0.2), the AUC (for the standard and recursive ALI), and both sensitivity and specificity (at a U threshold of 0.2 for the recursive ALI).

## Discussion

The present study aimed to demonstrate that brain tumors can be automatically identified, in an operator-independent way, from single MR images, using a modified version of a previously described procedure (Seghier et al., 2008). Our results show that this new procedure, which we refer to as recursive ALI, was able to delineate all the tumors at the right location, with high accuracy in 17 out of 18 different patients, despite the heterogeneous range of tumors included in our sample. The success of the tumor identification was validated in comparison to manual segmentation which is the current gold standard approach. We also show how tumor identification, in some patients, improved with the new recursive-ALI approach compared to the standard ALI approach. The recursive ALI procedure that we propose here will be particularly useful for studies of lesion-behavior mappings with large samples of patients. It may also have potential uses for surgical or diagnostic purposes.

Our recursive ALI procedure was able to identify tumors at the correct location in all the patients irrespective of the type, size or location. We stress here that the output was not determined by the particular low T1 signal in the tumors (as compared to the signal in the WM, see Figure 3). Successful automated delineation is also possible even if the tumors exhibited hypersignal because the critical point is not how darker or brighter the tumors appear in MRI scans but whether the signal is abnormal in comparison to what is typically expected at that particular location. It is also important to keep in mind that the outlier detection algorithm operates on the segmented GM and WM tissue classes rather than the raw T1 signal. Additionally, our method identified the extent of the tumor in all but one case (Patient 18 who had a high grade tumor, see below). The AUC from the ROC analysis was significantly higher for recursive ALI than for standard ALI. Notably, recursive ALI showed the biggest improvements when tumor delineation was challenging for the standard procedure (Patients 6 and 16). Patient 6 had extensive oedema near the inter-hemispheric fissure. Patient 16 had a tumor with a signal distribution close to the normal GM and located in the insula (Figure 2) where high inter-subject variability in normal anatomy limits the effectiveness of the segmentation priors. In sum, identifying brain tumors with recursive ALI is equal to or better than that obtained with standard ALI. Therefore, it may also be appropriate for the identification of other types of lesions that are problematic in the standard ALI implementation.

When comparing recursive ALI to the manually segmented tumors, the Dice similarity index (at a U threshold of 0.2) and the AUC were on average 0.72 and 0.97, respectively (See Figure 4 and Table 1). Therefore, both indices suggest an excellent agreement between automated and manual segmentations performed by each observer for the same tumors. To interpret our results, it is important to keep in mind the inherent inter-operator variability when using manual tracing as the gold standard method. By comparing manual lesion segmentation between and within observers, previous studies have shown non-negligible intra- and inter-operator variability for lesion identification (Fiez et al., 2000; Kaus et al., 2001). For instance, in our data, Dice's similarity index between the two independent observers was 0.838 on average, with higher variability across patients with diffuse signal (e.g., Patients 3 and 18, Table 1). In the same way, by defining variability as the percentage of non-overlapping voxels between two manually segmented lesions (cf. Fiez et al., 2000), the inter-operator variability was on average 27% (*SD* = 15%) in our data. This figure is comparable to the one reported by Fiez et al. (2000) at 33% (*SD* = 7%) in inter-operator variability between two observers segmenting MRI images from 10 patients with left frontal lesions. This indicates that (1) the definition of true positives and negatives is contaminated by subjectivity in the observers' manual segmentation. This would yield lower sensitivity and specificity for the recursive ALI method because, by definition, any alternative method cannot outperform the gold standard method and (2) our procedure would be a reasonable substitute for an observer, since the differences between the automated and the manual method are at a similar magnitude as the differences between observers, but with the advantage of being unbiased and replicable.

Recursive ALI may also detect secondary lesion effects such as atypical enlarged ventricles or widened sulci in the ipsi- and/or contra-lesional hemisphere. In our sample this was the case of Patient 2, in which enlarged ventricles were defined as lesion by the recursive ALI (see Figure 3). If these areas need to be omitted, then users can either (1) factor them out by including older healthy subjects who are likely to have visible aging-induced atrophy, or (2) adjust the threshold parameters when converting the fuzzy lesion image to the binary lesion image. The latter solution will only help, however, when the size of secondary effects are distant to and smaller than the primary damage. For example, in Patient 2, the ventricular dilations can easily be eliminated from the binary lesion image by increasing the cluster extent threshold. In the same way, the identification of tiny tumors (e.g., size <0.5 cm^3^) might be challenging because such small tumors express themselves at a spatial scale comparable to the expected normal variability in anatomy (few millimetres). As a general principle, for such challenging cases, we recommend all identified tumors be checked by eye.

**Figure 5:**
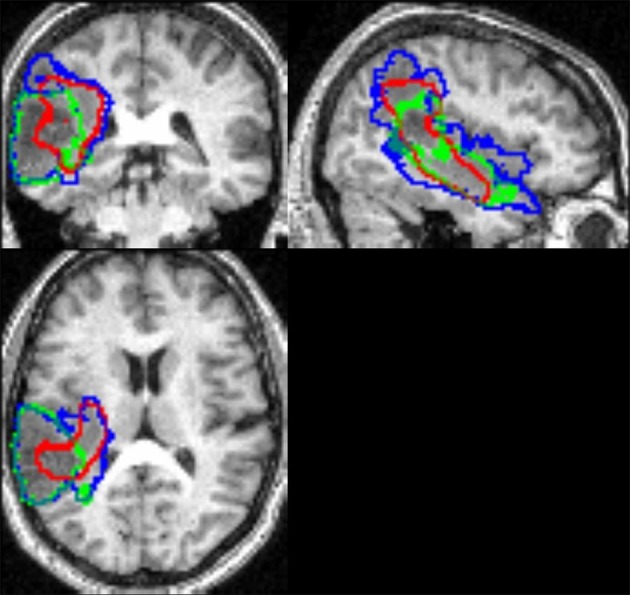
**The tumor contour for Patient 18 identified by recursive ALI in red, Observer 1 in blue, and Observer 2 in green.** This illustrates inconsistency between observers and the areas missed by the recursive ALI procedure (e.g., in the parieto-temporal junction).

Tumor identification in Patient 18 was the least successful (see Figure 4 and Table 1). This patient had a high grade tumor (glioblastoma) located in the temporo-parietal junction that infiltrated ventrally into the temporal pole and dorsally into the superior parietal lobule (Figure 5), resulting in an unclear border between intact and abnormal tissue. Consequently, the two observers disagreed on the exact borders of the tumor (see illustration in Figure 5), causing a relatively low Dice index (0.60). This situation is not an unusual occurrence in manual segmentation, even in studies with more than two observers (e.g., Kaus et al., 2001; Kubben et al., 2010). One possibility is to define the proper lesion as the set of voxels with the largest overlap over observers' manual segmentation, a rationale equivalent to “the consensus lesion” suggested previously by Fiez et al. (2000). By taking the overlap between the two observers' manual segmentation as the best approximation of Patient 18's tumor, we found that the segmentation step misclassified some of the tumor as “intact” GM even with the inclusion of the patient-specific prior (see red contours in Figure 5). Overall, the difficulty delineating the exact tumor site in Patient 18, irrespective of whether tumor identification was conducted automatically or manually, highlights (1) the importance of visual inspection following automated tumor identification and (2) the limitation of relying on a single MRI image. The inclusion of other brain images (e.g., contrast-enhanced images, FLAIR, T2-weighted or DWI images) may be required in these cases (see below). Alternatively, we recommend (1) adjusting the probability threshold of the extra prior in the iterative segmentation step to avoid misclassification of abnormal voxels as either GM or WM, (2) adjusting the parameters of the fuzzy clustering algorithm for outlier detection, or (3) repeating the recursive procedure more than twice to refine the patient specific prior for the extra class. Note, however, that in the latter case, multiple repetitions are time consuming and might also yield misclassification of intact tissue into the extra prior. For a discussion on the effects of the number of iterations and other parameters on the lesion identification algorithm see Seghier et al. (2007, 2008). These adjustments may be particularly important for our automated tumor identification which is expected to be problematic when there is an infiltrative tumor with a complex texture and unclear boundaries (Patient 18) or when tissue misclassification occurs in areas with high anatomical variability between controls (Patient 16).

Although the ALI procedure identifies lesions at the individual patient level, it is critically dependent on accurate spatial normalization. This is because the segmentation priors are in standard MNI space and also because the comparison of GM and WM images in the patient and controls during outlier detection relies on accurate warping of lesioned and healthy brains to the same standard reference space. The challenges of spatial normalization in brain images with lesions are well-documented (Brett et al., 2001; Crinion et al., 2007; Andersen et al., 2010; Ripollés et al., 2012). Our proposed recursive ALI procedure may help to improve spatial normalization of lesioned brains. Specifically, by optimizing an extra prior, at the individual patient level, the recursive ALI procedure may reduce tissue misclassification and thus may enhance the precision of the warping. This would ensure accurate spatial normalization, as implemented within the unified segmentation-normalization framework (Ashburner and Friston, 2005), yielding higher statistical power in anatomical and functional imaging studies of groups of patients with brain lesions.

The results of this study also offer another illustration of the flexibility of ALI when dealing with different types of brain damage. Indeed, modified versions of ALI have been successfully applied in clinical contexts, for instance in the identification of cerebral microbleeds from low-resolution T2^*^-weighted images (Seghier et al., 2011), the delineation of lesions from clinical CT scans (Chechlacz et al., 2012), and the detection of corticospinal tract damage in stroke patients (Kou et al., 2013). Its robustness on monospectral data makes ALI an attractive tool for other lesion types that can be fully characterized with a single imaging modality. This includes, for instance, the detection of white matter hyperintensities that typically appears on T2-weighted fluid attenuated inversion recovery (FLAIR) images (Wen and Sachdev, 2004), where automated identification (e.g., Wu et al., 2006a) of such white matter hyperintensities can be clinically useful in assessing risk factors of stroke and dementia (see meta-analysis in Debette and Markus, 2010).

Our point here is that the segmentation procedure used in ALI can be optimized for a given type of brain damage by explicitly incorporating any prior knowledge that can be provided by theoretical or other empirical data. For example, in the context of brain tumors, it is perfectly possible to define subject-specific priors that could be derived from other contrast images that are likely to be available in the clinical setting (e.g., contrast-enhanced images, FLAIR, T2-weighted or DWI images, see Prastawa et al., 2003), or even include more than one extra class to model the different types of the abnormal tissue (e.g., see Zacharaki et al., 2012). Indeed, the accuracy of ALI might be boosted by incorporating other contrast images because any additional information in those images may help the segmentation procedure to optimally differentiate between abnormal and normal brain tissue. When multi-contrast images are available, users may choose to run ALI (i.e., the segmentation step) in a multispectral mode, provided all contrast images are spatially co-registered and resliced. The effectiveness of multispectral segmentation in ALI for tumor identification warrants future investigations. An accurate delineation of the tumor can then be combined with pre-operative fMRI mapping of the same patients to support neurosurgical planning and enhance the efficiency and safety of the image-guided procedures (e.g., Tieleman et al., 2009; Kekhia et al., 2011).

Finally, although the output from our recursive ALI procedure is a fuzzy set indexing the degree of abnormality at each voxel of the brain, it is also possible to use the final segmented extra class as a proxy of the tumor that might be particularly useful for people interested in probabilistic estimates of tumors. We found very good correspondence between the segmented tissue in the extra class (i.e., the output of the segmentation during the second run of ALI) and the manual lesion segmentation of each patient (e.g., see Figure 2). The use of the fuzzy clustering algorithm, however, allows patient-specific tissue classes to be compared to a set of images from matched healthy controls (e.g., with similar demographics and/or acquisition protocols). Last but not least, we found that Dice indices were higher when the threshold on the degree of abnormality U ranged from 0.1 to 0.3 (Figure 4). For similar datasets, we thus recommend the use of U thresholds within this range if users are interested in generating binary definitions of their patients' tumors. This threshold needs to be increased (e.g., at 0.5) if a small number of controls were used in the outlier detection algorithm. As the case for any method that relies upon fuzzy or soft clustering techniques, the optimal threshold can be adjusted for a given dataset (see Shen et al., 2010 for a discussion).

To conclude, we have optimized our automated lesion identification procedure for brain tumors. This involved improving the tissue segmentation by including a data-driven recursive step that generated a patient-specific prior for the lesioned tissue. The rationale for developing this procedure was based on the need to identify tumors from monospectral MRI images when additional MRI contrast images are not available. Our findings demonstrate the success of the recursive ALI procedure by delineating different brain tumors with very good accuracy in a sample of 18 patients whose tumors differed in type, size and location. The recursive ALI procedure we introduce here therefore offers an objective and replicable method for brain tumor identification that will be relevant for lesion-behavior mapping. In addition, it may also be applied for the identification of other types of lesions that are problematic in the standard ALI implementation and may help to achieve accurate spatial normalization of brains with tumors for robust group analyses. The present paper also illustrates that ALI is a versatile tool that can be adapted for automated identification of any type of brain damage.

## Authors contributions

Ana Sanjuán, Cathy J. Price, and Tarek A. Yousry conceived and designed the experiment; Ana Sanjuán analyzed the data; Mohamed L. Seghier designed the toolbox used in analysis; Ana Sanjuán and Sharon Geva manually segmented the tumors; Laura Mancini, Goulven Josse, Alice Grogan, Adam K. Yamamoto, Alex P. Leff and Tarek A. Yousry contributed with patient selection and data acquisition; Ana Sanjuán, Cathy J. Price, and Mohamed L. Seghier wrote the paper; Ana Sanjuán, Cathy J. Price, Laura Mancini, Adam K. Yamamoto, Sharon Geva, Alex P. Leff, Tarek A. Yousry, and Mohamed L. Seghier revised the manuscript.

### Conflict of interest statement

The authors declare that the research was conducted in the absence of any commercial or financial relationships that could be construed as a potential conflict of interest.

## References

[B1] Andersen S. M. Rapcsak S. Z. Beeson P. M. (2010). Cost function masking during normalization of brains with focal lesions: still a necessity? Neuroimage 53, 78–84 10.1016/j.neuroimage.2010.06.003 20542122 PMC2938189

[B2] Ashburner J. Friston K. J. (2005). Unified Segmentation. Neuroimage 26, 839–851 10.1016/j.neuroimage.2005.02.018 15955494

[B3] Asman A. J. Chambless L. B. Thompson R. C. Landman B. A. (2013). Out-Of-Atlas likelihood estimation using multi-atlas segmentation. Med. Phys. 40, 043702 10.1118/1.4794478 23556928 PMC3625241

[B4] Assefa D. Keller H. Ménard C. Laperriere N. Ferrari R. J. Yeung I. (2010). Robust texture features for response monitoring of glioblastoma multiforme on T1-weighted and T2-FLAIR MR images: a preliminary investigation in terms of identification and segmentation. Med. Phys. 37, 1722–1736 10.1118/1.3357289 20443493

[B5] Bates E. Wilson S. M. Saygin A. P. Dick F. Martin I. S. Knight R. T. (2003). Voxel-based lesion-symptom mapping. Nat. Neurosci. 6, 448–450 10.1038/nn1050 12704393

[B6] Brett M. Leff A. P. Rorden C. Ashburner J. (2001). Spatial normalization of brain images with focal lesions using cost function masking. Neuroimage 14, 486–500 10.1006/nimg.2001.0845 11467921

[B7] Capelle A. S. Colot O. Fernandez-Maloigne C. (2004). Evidential segmentation scheme of multi-echo MR images for the detection of brain tumors using neighborhood information. Inf. Fusion 5, 203–216 10.1016/j.inffus.2003.10.001

[B8] Cardoso M. J. Clarkson M. J. Ridgway G. R. Modat M. Fox N. C. Ourselin S. (2011). LoAd: a locally adaptive cortical segmentation algorithm. Neuroimage 56, 1386–1397 10.1016/j.neuroimage.2011.02.013 21316470 PMC3554791

[B9] Chechlacz M. Rotshtein P. Roberts K. L. Bickerton W. L. Lau J. K. Humphreys G. W. (2012). The prognosis of allocentric and egocentric neglect: evidence from clinical scans. PLoS ONE 7:e47821 10.1371/journal.pone.0047821 23133604 PMC3486857

[B10] Corso J. J. Sharon E. Dube S. El-Saden S. Sinha U. Yuille A. (2008). Efficient multilevel brain tumor segmentation with integrated bayesian model classification. IEEE Trans. Med. Imaging 27, 629–640 10.1109/TMI.2007.912817 18450536

[B11] Crinion J. Ashburner J. Leff A. Brett M. Price C. Friston K. (2007). Spatial normalization of lesioned brains: performance evaluation and impact on fMRI analyses. Neuroimage 37, 866–875 10.1016/j.neuroimage.2007.04.065 17616402 PMC3223520

[B12] Debette S. Markus H. S. (2010). The clinical importance of white matter hyperintensities on brain magnetic resonance imaging: systematic review and meta-analysis. BMJ 341:c3666 10.1136/bmj.c3666 20660506 PMC2910261

[B13] Deichmann R. Schwarzbauer C. Turner R. (2004). Optimisation of the 3D MDEFT sequence for anatomical brain imaging: technical implications at 1.5 and 3 T. Neuroimage 21, 757–767 10.1016/j.neuroimage.2003.09.062 14980579

[B14] Dice L. R. (1945). Measures of the amount of ecological association between species. Ecology 26, 297–302 10.2307/193240916136810

[B15a] Fiez J. A. Damasio H. Grabowski T. J. (2000). Lesion segmentation and manual warping to a reference brain: intra- and interobserver reliability. Hum. Brain Mapp. 9, 192–211 10.1002/(SICI)1097-0193(200004)9:4<192::AID-HBM2>3.0.CO;2-Y 10770229 PMC6871916

[B15] Geva S. Baron J. C. Jones P. S. Price C. J. Warburton E. A. (2012). A comparison of VLSM and VBM in a cohort of patients with post-stroke aphasia. Neuroimage 1, 37–47 10.1016/j.nicl.2012.08.003 24179735 PMC3757730

[B16] Gondal A. H. Khan M. N. A. (2013). A review of fully automated techniques for brain tumor detection from MR images. I. J. Mod. Educ. Comput. Sci. 2, 55–61 10.5815/ijmecs.2013.02.08

[B17] Gordillo N. Montseny E. Sobrevilla P. (2013). State of the art survey on MRI brain tumor segmentation. Magn. Reson. Imaging 31, 1426–1438 10.1016/j.mri.2013.05.002 23790354

[B18] Hanley J. A. McNeil B. J. (1982). The meaning and use of the area under a receiver operating characteristic (ROC) curve. Radiology 143, 29–36 7063747 10.1148/radiology.143.1.7063747

[B19] Harati V. Khayati R. Farzan A. (2011). Fully automated tumor segmentation based on improved fuzzy connectedness algorithm in brain MR images. Comput. Biol. Med. 41, 483–92 10.1016/j.compbiomed.2011.04.010 21601840

[B20] Hsieh T. M. Liu Y. Liao C.-C. Xiao F. Chiang I. Wong J. (2011). Automatic segmentation of meningioma from non-contrasted brain MRI integrating fuzzy clustering and region growing. BMC Med. Inform. Decis. Mak. 11:54 10.1186/1472-6947-11-54 21871082 PMC3189096

[B21] Ibrahim A. M. (1996). Introduction to Applied Fuzzy Electronics. Lebanon, IN: Prentice Hall

[B22] Kabir Y. Dojat M. Scherrer B. Forbes F. Garbay C. (2007). Multimodal MRI segmentation of ischemic stroke lesions, in 29th Annual International Conference of the IEEE Engineering in Medicine and Biology Society EMBC (Lyon). 10.1109/IEMBS.2007.4352610 PMC2735153 18002276

[B23] Kaus M. R. Warfield S. K. Nabavi A. Black P. M. Jolesz F. A. Kikinis R. (2001). Automated segmentation of MR images of brain tumors. Radiology 218, 586–591 10.1148/radiology.218.2.r01fe44586 11161183

[B24] Kekhia H. Rigolo L. Norton I. Golby A. J. (2011). Special surgical considerations for functional brain mapping. Neurosurg. Clin. N. Am. 22, 111–132 10.1016/j.nec.2011.01.004 21435565 PMC3064825

[B25] Kou N. Park C-H. Seghier M. L. Leff A. P. Ward N. S. (2013). Can fully automated detection of corticospinal tract damage be used in stroke patients? Neurology 80, 2242–2245 10.1212/WNL.0b013e318296e977 23658388 PMC3721100

[B26] Kubben P. L. Postma A. A. Kessels A. G. van Overbeeke J. J. van Santbrink H. (2010). Intraobserver and interobserver agreement in volumetric assessment of glioblastoma multiforme resection. Neurosurgery 67, 1329–1334 10.1227/NEU.0b013e3181efbb08 20871451

[B27] Levy-Cooperman N. Ramirez J. Lobaugh N. J. Black S. E. (2008). Misclassified tissue volumes in Alzheimer disease patients with white matter hyperintensities: importance of lesion segmentation procedures for volumetric analysis. Stroke 39, 1134–1141 10.1161/STROKEAHA.107.498196 18323507

[B28] Lladó X. Oliver A. Cabezas M. Freixenet J. Vilanova J. Quiles A. (2012). Segmentation of multiple sclerosis lesions in brain MRI: a review of automated approaches Information Sciences 1, 164–185 10.1016/j.ins.2011.10.011

[B29] Lobo J. M. Jiménez-Valverde A. Real R. (2008). AUC: a misleading measure of the performance of predictive distribution models. Global Ecol. Biogeogr. 17, 145–151 10.1111/j.1466-8238.2007.00358.x

[B30] Menze B. H. Van Leemput K. Lashkari D. Weber M. A. Ayache N. Golland P. (2010). A generative model for brain tumor segmentation in multi-modal images. Med. Image Comput. Comput. Assist. Interv. 13(Pt 2), 151–159 10.1007/978-3-642-15745-5_19 20879310 PMC3050038

[B31] Metz C. E. (1978). Basic principles of ROC analysis. Semin. Nucl. Med. 8, 283–298 10.1016/S0001-2998(78)80014-2 112681

[B32] Nie J. Xue Z. Liu T. Young G. S. Setayesh K. Guo L. Wong S. T. (2009). Automated brain tumor segmentation using spatial accuracy-weighted hidden Markov Random Field. Comput. Med. Imaging Graph. 33, 431–441 10.1016/j.compmedimag.2009.04.006 19446435 PMC2739047

[B33] Popuri K. Cobzas D. Murtha A. Jägersand M. (2012). 3D variational brain tumor segmentation using Dirichlet priors on a clustered feature set. Int. J. Comput. Assist. Radiol. Surg. 7, 493–506 10.1007/s11548-011-0649-2 21833491

[B34] Powers D. M. W. (2012). The problem of area under the curve, in International Conference on Information Science and Technology (ICIST) IEEE (Piscataway, NJ).

[B35] Prastawa M. Bullitt E. Ho S. Gerig G. (2004). A brain tumor segmentation framework based on outlier detection. Med. Image Anal. 8, 275–283 10.1016/j.media.2004.06.007 15450222

[B36] Prastawa M. Bullitt E. Moon N. Van Leemput K. Gerig G. (2003). Automatic brain tumor segmentation by subject specific modification of atlas priors. Acad. Radiol. 10, 1341–1348 10.1016/S1076-6332(03)00506-3 14697002 PMC2430604

[B37] Ripollés P. Marco-Pallarés J. de Diego-Balaguer R. Miró J. Falip M. Juncadella M. (2012). Analysis of automated methods for spatial normalization of lesioned brains. Neuroimage 60, 1296–1306 10.1016/j.neuroimage.2012.01.094 22305954

[B38] Rorden C. Fridriksson J. Karnath H. O. (2009). An evaluation of traditional and novel tools for lesion behaviour mapping. Neuroimage 44, 1355–1362 10.1016/j.neuroimage.2008.09.031 18950719 PMC2667945

[B39] Rouainia M. Medjram M. S. Doghmane N. (2006). Brain MRI segmentation and lesions detection by EM algorithm. Proc. World Acad. Sci. Eng. Technol. 17, 301–304 12060018

[B40] Ruan S. Lebonvallet S. Merabet A. Constans J. M. (2007). Tumor segmentation from a multispectral MRI images by using support vector machine classification, in IEEE International Symposium on Biomedical Imaging, (Arlington, VA), 1236–1239

[B41] Seghier M. L. Friston K. J. Price C. J. (2007). Detecting subject-specific activations using fuzzy clustering Neuroimage 36, 594–605 10.1016/j.neuroimage.2007.03.021 17478103 PMC2724061

[B42] Seghier M. L. Kolanko M. A. Leff A. P. Jäger H. R. Gregoire S. M. Werring D. J. (2011). Microbleed detection using automated segmentation (MIDAS): a new method applicable to standard clinical MR images. PLoS ONE 6:e17547 10.1371/journal.pone.0017547 21448456 PMC3063172

[B43] Seghier M. L. Ramlackhansingh A. Crinion J. Leff A. P. Price C. J. (2008). Lesion identification using unified segmentation-normalisation models and fuzzy clustering. Neuroimage 41, 1253–1266 10.1016/j.neuroimage.2008.03.028 18482850 PMC2724121

[B44] Shen S. Szameitat A. J. Sterr A. (2008). Detection of infarct lesions from single MRI modality using inconsistency between voxel intensity and spatial location-a 3-D automatic approach. IEEE Trans. Inf. Technol. Biomed. 12, 532–540 10.1109/TITB.2007.911310 18632333

[B45] Shen S. Szameitat A. J. Sterr A. (2010). An improved lesion detection approach based on similarity measurement between fuzzy intensity segmentation and spatial probability maps. Magn. Reson. Imaging 28, 245–254 10.1016/j.mri.2009.06.007 19695812

[B46] Soltanian-Zadeh H. Peck D. J. Windham J. P. Mikkelsen T. (1998). Brain tumor segmentation and characterization by pattern analysis of multispectral NMR images. NMR Biomed. 11, 201–208 10.1002/(SICI)1099-1492(199806/08)11:4/5<201::AID-NBM508>3.0.CO;2-6 9719574

[B47] Thesen T. Quinn B. T. Carlson C. Devinsky O. DuBois J. McDonald C. R. (2011). Detection of epileptogenic cortical malformations with surface-based MRI morphometry. PLoS ONE 6:16430 10.1371/journal.pone.0016430 21326599 PMC3033882

[B48] Tieleman A. Deblaere K. Van Roost D. Van Damme O. Achten E. (2009). Preoperative fMRI in tumour surgery. Eur. Radiol. 19, 2523–2534 10.1007/s00330-009-1429-z 19430795

[B49] Veloz A. Orellana A. Vielma J. Salas R. Chabert S. (2011). Brain tumours: How can images and segmentation techniques help?, in Diagnostic Techniques and Surgical Management of Brain Tumors, ed Abujamra A. L. (InTech), 67–92 10.5772/22466 Available online at: http://www.intechopen.com/books/diagnostic-techniques-and-surgical-management-of-brain-tumors/brain-tumors-how-can-images-and-segmentation-techniques-help-

[B50] Wen W. Sachdev P. (2004). The topography of white matter hyperintensities on brain MRI in healthy 60- to 64-year-old individuals. Neuroimage 22, 144–154 10.1016/j.neuroimage.2003.12.027 15110004

[B51] Wu M. Rosano C. Butters M. Whyte E. Nable M. Crooks R. (2006a). A fully automated method for quantifying and localizing white matter hyperintensities on MR images. Psychiatry Res. 148, 133–142 10.1016/j.pscychresns.2006.09.003 17097277 PMC1761950

[B52] Wu Y. Warfield S. K. Tan I. L. Wells W. M. Meier D. S. van Schijndel R. A. (2006b). Automated segmentation of multiple sclerosis lesion subtypes with multichannel MRI. Neuroimage 32, 1205–1215 10.1016/j.neuroimage.2006.04.211 16797188

[B53] Xu T. Mandal M. (2012). Automatic brain tumor extraction from T1-Weighted coronal mri usding fast bounding box and dynamic snake. Conf. Proc. IEEE Eng. Med. Biol. Soc. 2012, 444–447 23365924 10.1109/EMBC.2012.6345963

[B54] Zacharaki E. Erus G. Bezerianos A. Davatzikos C. (2012). Fuzzy Multi-channel clustering with individualized spatial priors for segmenting brain lesions and infarcts, in Artificial Intelligence Applications and Innovations. In IFIP Advances in Information and Communication Technology, Vol. 382, (Halkidiki: Springer Berlin Heidelberg), 76–85 10.1007/978-3-642-33412-2_8

[B55] Zou K. H. Wells W. M. 3rd Kikinis R. Warfield S. K. (2004). Three validation metrics for automated probabilistic image segmentation of brain tumours. Stat. Med. 23, 1259–1282 10.1002/sim.1723 15083482 PMC1463246

